# Effects of education level on natural language processing in cardiovascular health communication

**DOI:** 10.3389/fpubh.2025.1688173

**Published:** 2025-11-13

**Authors:** Stanley Joseph, Ashna Bhardwaj, Justin Skariah, Ishan Aggarwal, Varunil Shah, Ryan A. Harris

**Affiliations:** 1 Medical College of Georgia, Augusta University, Augusta, GA, United States; 2 Case Western Reserve University School of Medicine, Cleveland, OH, United States; 3 Georgia Prevention Institute, Augusta University, Augusta, GA, United States

**Keywords:** cardiovascular disease, artificial intelligence, natural language processing, health communication, readability, patient education, large language models

## Abstract

**Introduction:**

Cardiovascular disease (CVD) remains the leading cause of mortality worldwide, underscoring the importance of accessible health communication. Artificial intelligence (AI) tools such as ChatGPT and MediSearch have potential to bridge knowledge gaps, but their effectiveness depends on both accuracy and readability. This study evaluated how natural language processing (NLP) models respond to CVD-related questions across different education levels.

**Methods:**

Thirty-five frequently asked questions from reputable sources were reformatted into prompts representing lower secondary, higher secondary, and college graduate levels, and entered into ChatGPT Free (GPT-4o mini), ChatGPT Premium (GPT-4o), and MediSearch (v1.1.4). Readability was assessed using Flesch–Kincaid Ease and Grade Level scores, and response similarity was evaluated with BERT-based cosine similarity. Statistical analyses included ANOVA, Kruskal-Wallis, and Pearson correlation.

**Results:**

Readability decreased significantly with increasing education level across all models (*p* < 0.001). ChatGPT Free responses were more readable than MediSearch (*p* < 0.001), while ChatGPT Free and Premium demonstrated higher similarity to each other than to MediSearch. ChatGPT Premium explained the greatest variance in readability (*r* = 0.350; *p* < 0.001), suggesting stronger adaptability to user education levels compared to ChatGPT Free (*r* = 0.530; *p* < 0.001) and MediSearch (*r* = 0.227; *p* < 0.001).

**Discussion:**

These findings indicate that while NLP models adjust readability by education level, output complexity often exceeds average literacy, highlighting the need for refinement to optimize AI-driven patient education.

## Introduction

Cardiovascular disease (CVD) is the leading cause of mortality worldwide, accounting for one in every five deaths. In 2022, CVD claimed approximately 700,000 lives in the United States, with treatment and management costs exceeding $250 billion (1). Effective CVD management and prevention depend heavily on public awareness, which is shaped by the availability of accurate and accessible health information from a variety of sources, including online searches. The growing prevalence of artificial intelligence (AI) and natural language processing (NLP) models is transforming healthcare, particularly by delivering personalized health advice to patients with cardiovascular conditions. NLP is a methodology that utilizes computer science, linguistics, and AI to understand, process, and interpret human languages (2). ChatGPT and MediSearch are a subset of NLPs called large language models (LLM), which generate human language based on user inputs (3).

In 2024, nearly a quarter of the U.S. population used ChatGPT for online health information (4). AI-driven tools like ChatGPT and MediSearch help bridge knowledge gaps by providing instant, tailored answers to health-related queries. Additionally, AI tools such as ChatGPT can serve as a reliable source of CVD information and could be recommended as supplementary resources to patients by cardiologists based on a 2024 study which determined the high degree of clinical accuracy (5). However, despite the growing use of AI, it is important to emphasize that AI-generated responses are not always accurate and interpretable by the user. In fact, AI-generated responses to questions related to atrial fibrillation were deemed “perfect” in only 4.7% of cases (6). Without physician oversight, maintaining high standards for these NLP models is crucial to ensure optimal delivery of information and patient care. While accuracy is a key focus, readability is equally important. If the health-related text output is too complex, it can hinder the user’s understanding and compromise decision-making, self-care, and subsequent outcomes, regardless of its accuracy. “Previous studies have also observed that ChatGPT’s responses in delivering answers to FAQs about heart failure educational material are often longer and more challenging to read compared to other resources, such as pamphlets and infographics (7). Recent investigations across other domains echo these findings: chatbot responses in cardiovascular, oncology, and dermatology contexts frequently exceeded recommended readability levels (8); coronary artery disease evaluations revealed inconsistent accuracy and accessibility (9); and ChatGPT’s performance in answering layperson questions on cardiac arrest demonstrated variability in clarity and appropriateness (10). More broadly, large language models have been shown to misalign with established readability standards, raising concerns about their suitability for diverse patient populations (11).

Collectively, these studies highlight the growing importance of evaluating not only the accuracy but also the accessibility of AI-generated health information. While prior work has emphasized that AI-generated responses often achieve high readability, these analyses have not systematically accounted for user education level when evaluating response quality. The proposed investigation fills a knowledge gap in the existing literature by shifting the lens from general readability toward user-specific adaptability. This framing highlights the novelty of our contribution within the cardiovascular disease domain. Despite the technical improvements in AI, a critical challenge remains with the use and implementation of this rapidly advancing technology. Therefore, ensuring that NLP-generated responses are not only accurate, but also comprehensible to users with varying education levels is necessary and has yet to be investigated. Thus, the present investigation evaluated how NLP models, such as ChatGPT and MediSearch, respond to cardiovascular disease-related questions across different educational levels. Specifically, how a user’s education level influences response readability and similarity across different NLPs was investigated. We hypothesized that as user education level increases, the readability of NLP-generated responses will decrease, and the similarity of responses across different models will decline.

## Materials and methods

### Question development

Frequently asked questions (FAQs) about various cardiovascular diseases were sourced from reputable references, covering conditions such as myocardial infarction (12–14), peripheral artery disease (15, 16), abdominal aortic aneurysm (17), stroke (18, 19), heart failure (20), coronary artery disease (21), and hypertension (22–25). Five FAQs were selected for each condition—35 questions in total—and restructured into a prompt-like format to simulate user input in an NLP model. While this number does not encompass the full scope of possible patient queries, it was intentionally chosen to balance thematic breadth and manageability across conditions while allowing for meaningful comparison across models. All prompts were entered manually into each model without any custom instructions, memory settings, or system-level prompt modifications. Default settings were used for ChatGPT Free, ChatGPT Premium, and MediSearch to ensure consistency. To minimize potential bias, all prompts were standardized using consistent formatting rules (e.g., phrased as patient questions, no additional qualifiers). Each of the 35 FAQs were reformulated into exactly three variants (one per education tier), yielding balanced distribution across lower secondary, higher secondary, and college graduate levels. Each prompt was assessed using the Flesch–Kincaid score to classify it into one of three educational levels: Lower Secondary Education (5th–8th grade), Higher Secondary Education (9th grade–high school graduate or GED equivalent), or College Graduate (bachelor’s degree or equivalent) (26). The prompts were then entered into ChatGPT Free (GPT-4o mini), ChatGPT Premium (GPT-4o), and MediSearch (version 1.1.4) to replicate the user/patient experience. The data was collected between August and December 2024. Although LLM outputs are stochastic, a single-run design was performed to reflect real-world patient use, where most users query once rather than multiple times. This pragmatic approach reduces burden while capturing the typical user experience. Indeed, future work should incorporate repeated runs to assess variability. Additionally, although 35 prompts may not fully capture the diversity of all cardiovascular disease queries, they serve as a representative sample for initial evaluation of model performance. Future studies with expanded datasets will be needed to assess broader generalizability. For reproducibility, 35 FAQs were each reformulated into three education-level variants. This resulted in 105 total prompts across 7 cardiovascular conditions (MI, PAD, AAA, Stroke, HF, CAD, and HTN). Distribution was balanced: 35 Easy, 35 Medium, and 35 Hard prompts. All prompts were manually entered once per model (ChatGPT Free, ChatGPT Premium, MediSearch) between August–December 2024. A single-run design was chosen, reflecting a pragmatic patient-query scenario, and full prompt text with Flesch–Kincaid scores is provided in Supplementary material.

### Readability scoring—Flesch–Kincaid Ease Score

Each response was evaluated based on the Flesch–Kincaid Ease Score. The Flesch–Kincaid Ease Score assigns a numerical value between 1 and 100, with higher scores indicating easier-to-read text (26). This metric has been extensively applied in both NLP and medical literature to evaluate text complexity and accessibility (27, 28). The following equation calculates the Flesch–Kincaid Ease Score (26):


$$\begin{array}{l} \text{Ease Score}=206.835-1.015(\frac{\text{Total Words}}{\text{Total Sentences}})\\ -84.6(\frac{\text{Total Syllables}}{\text{Total Words}}) \end{array}$$

### Readability scoring—Flesch–Kincaid Grade Level and Flesch–Kincaid Reading Level

The Flesch–Kincaid Grade Level provides a numerical assessment from 0 to 18, where higher scores indicate more complex text. It has been frequently utilized in NLP and healthcare research to quantify readability and ensure accessibility in medical communications (5, 6, 27, 28). This metric also aligns with the U.S. education system and serves as the basis for the Flesch–Kincaid Reading Level, which categorizes text complexity into educational grade levels (e.g., 8th grade, 10th grade, college, or college graduate). The following equation calculates the Flesch–Kincaid Grade Level (22):


$$\begin{array}{l} \text{Grade Level}=0.39(\frac{\text{Total Words}}{\text{Total Sentences}})\\ -11.8(\frac{\text{Total Syllables}}{\text{Total Words}})-15.59 \end{array}$$

### Similarity scoring

Once each response was recorded, they were compared using a Bidirectional Encoder Representations from Transformers (BERT) embedding technique combined with a Cosine Similarity model, a widely used method in natural language processing for measuring semantic similarity between text representations (29). Specifically, the sentence-transformers/all-MiniLM-L6-v2 model (384-dimensional embeddings) was used, implemented in Python with the sentence-transformers package (v2.2.2). Minimal preprocessing was performed (lowercasing, punctuation preserved), and embeddings were aggregated using mean pooling across tokens. No dimensionality reduction was applied. BERT converts text into high-dimensional vectors, and the similarity between two sets of text is measured by the cosine of the angle between their respective vectors. This results in a similarity score ranging from 0 to 1, where scores closer to 1 correspond to higher similarity and indicate more consistent responses across models while scores closer to 0 suggest greater variability (30). Comparisons were conducted between ChatGPT Free and ChatGPT Premium, ChatGPT Free and MediSearch, and ChatGPT Premium and MediSearch.

### Statistical analysis

To determine statistically significant differences in readability among education levels, a univariate ANOVA was applied to normally distributed data, while a Kruskal-Wallis test was used for non-normally distributed data. Normality was assessed using the Shapiro–Wilk test. A two-factor ANOVA with a Tukey *post-hoc* test was conducted to evaluate the effects of education level and NLP model type on both readability and similarity. Additionally, a Pearson Correlation was performed to evaluate all potential relationships among readability, similarity, and education level. Additionally, because FK scores are influenced by response length, both word count and sentence count were controlled by using a prompt-level mixed-effects model (random intercept for prompt). Values in this study are presented as mean ± standard deviation, unless otherwise noted. Modeling was developed in Python (version 3.12.5), and all statistical analyses were performed using R (version 4.3.1) and GraphPad Prism (version 10.4.1). Significance was set at *p* < 0.05.

## Results

### Readability scoring

**Figure 1:**
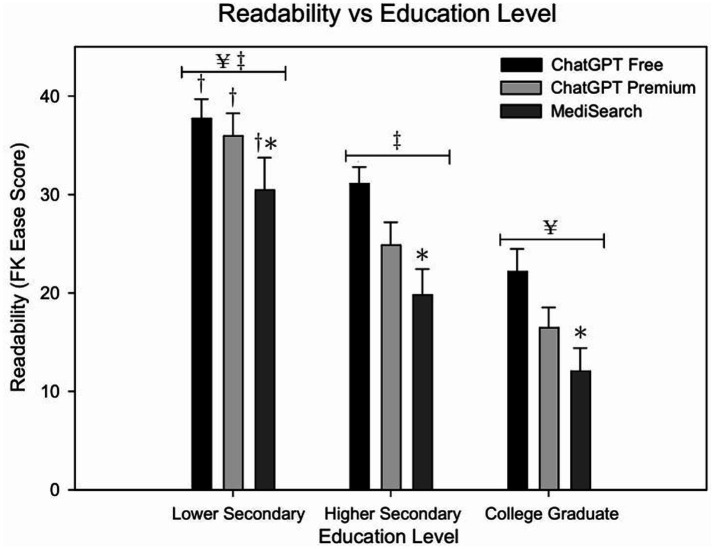
Readability of AI-generated responses across education levels. Mean Flesch–Kincaid Ease Scores for each AI model across education level. Error bars represent the standard error of the mean (SEM) with significant relationships marked by symbols: (*) indicates significance from ChatGPT Free within education level; (†) indicates significance from higher secondary education within tool; (¥) indicates significance from higher secondary education collapsing across education levels; (‡) indicates significance from college graduate collapsing across education levels.

**Figure 2:**
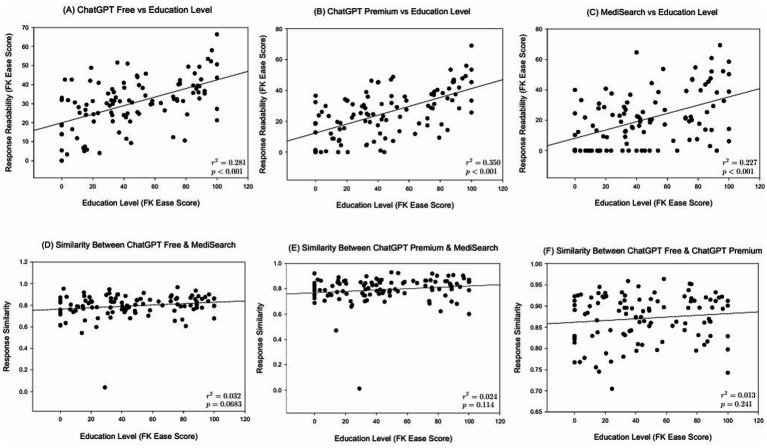
Correlations between education level and both readability and similarity metrics. Scatter plot illustrating the relationship between education level and **(A)** the Flesch–Kincaid Ease Score of ChatGPT Free responses, **(B)** the Flesch–Kincaid Ease Score of ChatGPT Premium responses, **(C)** the Flesch–Kincaid Ease Score of responses generated by MediSearch, **(D)** similarity scores between ChatGPT Free and MediSearch, **(E)** similarity scores between responses generated by ChatGPT Premium and MediSearch, and **(F)** similarity scores between responses from ChatGPT Premium and ChatGPT Free.

Figure 1 illustrates the mean Flesch–Kincaid Ease Score by education level and model. ChatGPT Free responses are significantly different when the user had a lower secondary education versus a college education (*p* < 0.001). Figure 2 illustrates the relationship between education level and both readability and similarity outcomes for each model. This difference was also seen in both ChatGPT Premium (*p* < 0.001) and MediSearch (*p* < 0.001). Additionally, readability changes between models. Specifically, there was a statistically significant difference in the readability of the responses between models for ChatGPT Premium (*p* = 0.029) and MediSearch (*p* = 0.043) when the user had lower secondary education versus higher secondary education. A significant positive relationship was observed between the Flesch–Kincaid Ease Score and education level for ChatGPT Free (Figure 2A; *r* = 0.530, *p* < 0.001), ChatGPT Premium (Figure 2B; *r* = 0.592, *p* < 0.001), and MediSearch (Figure 2C; *r* = 0.227, *p* = 0.017) (see Tables 1, 2).

**Table 1:** Readability of AI-generated responses across education levels.

Education level	ChatGPT Premium (*n* = 35)	ChatGPT Free (Mini) (*n* = 35)	MediSearch (*n* = 35)
Lower secondary	35.95 ± 13.63	37.72 ± 11.67	30.45 ± 19.55
Higher secondary	24.85 ± 13.84	31.11 ± 10.01	19.80 ± 15.57
College graduate	16.47 ± 12.20	22.16 ± 13.71	12.05 ± 13.95

Mean readability scores (± SD) for ChatGPT Premium, ChatGPT Free (Mini), and MediSearch across lower secondary, higher secondary, and college graduate education levels. Higher values indicate responses easier to read.

**Table 2:** Textual similarity of AI-generated responses across education levels.

Education level	Premium vs MS (*n* = 35)	Mini vs MS (*n* = 35)	Premium vs Mini (*n* = 35)
Lower secondary	0.815 ± 0.081	0.818 ± 0.078	0.878 ± 0.050
Higher secondary	0.804 ± 0.059	0.806 ± 0.069	0.876 ± 0.048
College graduate	0.759 ± 0.155	0.755 ± 0.158	0.859 ± 0.067

Mean similarity indices (± SD) for pairwise comparisons between ChatGPT Premium, ChatGPT Free (Mini), and MediSearch (Premium vs MediSearch, Mini vs MediSearch, Premium vs Mini) across lower secondary, higher secondary, and college graduate education levels. Values closer to 1 indicate greater textual overlap between models.

### Similarity scoring

**Figure 3:**
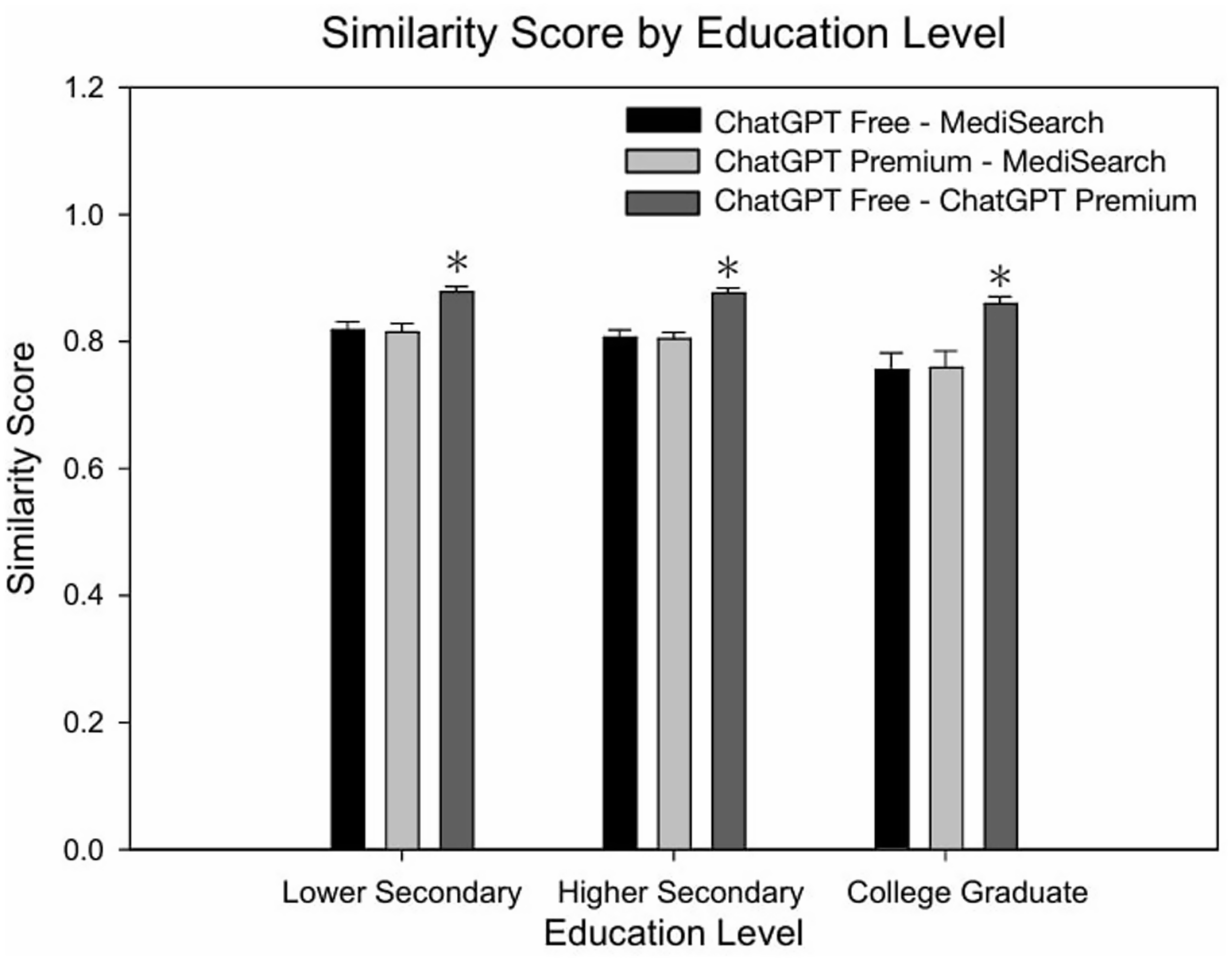
Similarity of AI-generated responses across education levels. Mean similarity scores for each AI model comparison across education levels. Error bars represent the standard error of the mean (SEM) with significant relationships marked by the symbol: (*) indicating significance from lower secondary and higher secondary within education level.

Figure 3 depicts the mean similarity score by education level which further exemplifies the similarity between both ChatGPT models versus the decreased similarity between either ChatGPT model with MediSearch, consistent across education levels. Specifically, the similarity between ChatGPT Free and MediSearch differed significantly from the similarity between ChatGPT Free and ChatGPT Premium for users with lower secondary education (*p* = 0.002), higher secondary education (*p* < 0.001), and college graduate education (*p* = 0.005). Additionally, the similarity between ChatGPT Premium and MediSearch was statistically different from the similarity between ChatGPT Free and ChatGPT Premium for users with lower secondary education (*p* < 0.001), higher secondary education (*p* < 0.001) and college graduate education (*p* = 0.006). In contrast, no statistical significant correlations between education level and the similarity between ChatGPT Free versus MediSearch (Figure 2D; *r* = 0.161, *p* < 0.102), ChatGPT Premium versus MediSearch (Figure 2E; *r* = 0.144, *p* < 0.136), nor ChatGPT Premium versus ChatGPT Free (Figure 2F; *r* = 0.104, *p* = 0.291) were observed.

In a supplementary analysis adjusting for word count and sentence count, readability differences between models persisted. MediSearch remained harder to read compared to ChatGPT Free by ~11 FK points (*p* < 0.001), and Premium harder by ~5 FK points (*p* < 0.001). Word count was negatively associated with readability (*p* = 0.001), while sentence count was positively associated (*p* < 0.001).

## Discussion

Cardiovascular disease remains a leading cause of morbidity and mortality worldwide, emphasizing the need for clear and accessible educational resources to enhance health outcomes. The overall use of AI is increasing at a rapid pace and has begun to enter the medical and health care space. The overall goal of the present investigation was to examine how three NLP models, ChatGPT (Free and Premium versions) and MediSearch, respond to user questions regarding CVD and investigate if the education level impacts the analyzing trends in readability and the similarity of their responses. The findings of the present investigation demonstrates that as the education level of the user increases, the readability of the NLP model response decreases, which has yet to be demonstrated within the cardiology domain across various pathologies. In addition, ChatGPT Premium is more effective at assessing users’ education levels and tailoring responses that align with the users understanding compared to ChatGPT Free and MediSearch. No identifiable relationship exists between education level and similarity between any pairwise comparisons between models. However, the similarity of the text differed when comparing MediSearch responses with either ChatGPT model.

### Readability of NLP responses to frequently asked CVD questions

Readability is a measure of sentence length and word complexity that evaluates how difficult a piece of writing is to read, often captured as a Flesch–Kincaid Ease Score or Grade Level (22). Findings of the present investigation identified several key relationships between readability, education level, and NLP model type. Specifically, as education level increases the mean Flesch–Kincaid Ease Score decreases for all models, indicating that increasing education level results in the text being more difficult to read (Figure 1). This data coincides with another study that determined large language models implicitly determine the text difficulty for users (31). Similar methods evaluating AI-generated cardiovascular or educational content have been reported in multiple recent studies using the Flesch–Kincaid or similar metrics for readability assessment (6–8, 10, 24), further validating the analytic approach used here. It is important to emphasize that as NLP models become more widely used, users can expect these models to adapt to their needs based on their underlying educational background.

Despite the models’ ability to adjust readability based on education levels, the high Flesch–Kincaid Grade Levels across all models suggest a potential accessibility barrier for individuals with lower education levels. This is particularly concerning considering the average reading level in the U.S. is 7th to 8th grade with 54% of American adults reading below a 6th grade level (32). Notably, the present study identified that each model produced responses that were at least a college graduate reading level. The high education level response may present an obstacle for individuals with lower education levels as complex medical language can certainly hinder comprehension, potentially leading to misunderstandings and improper self-management of cardiovascular conditions. Previous studies align with present study’s readability findings, further supporting that models like ChatGPT produce responses exceeding the grade-level recommendations set by the National Institutes of Health and the American Medical Association when addressing cardiovascular disease queries (6). Indeed, ChatGPT is most widely used; however, a similar observation was observed with MediSearch, as it generated even more complex responses on average compared to ChatGPT. The significant positive correlation between user education level and readability suggests that all NLP models adapt responses to match perceived user education. Among these, ChatGPT Premium most robustly aligns readability with user education level, potentially offering enhanced tailored responses.

While each model generally produced complex responses, ChatGPT Free was easier to understand compared to MediSearch (*p* < 0.001). Given that ChatGPT Free is a widely accessible, no-cost tool, this finding suggests that it may be better suited for the general public, particularly individuals with lower education levels who may not want—or be able—to invest in a premium AI service like ChatGPT Premium. Although MediSearch is also free, its higher complexity could make it less practical for broad public use, especially for those seeking easily digestible healthcare information. Furthermore, while no concrete data on usage trends is available, it appears that ChatGPT Free is more widely used, reinforcing its potential as a more effective tool for cardiovascular disease queries.

It is worth noting that differences in response length across models may have influenced readability scores. For instance, MediSearch responses tended to be longer and potentially more complex, which may have contributed to lower Flesch–Kincaid Ease Scores. This factor should be considered when interpreting readability differences. Although longer responses were indeed associated with lower readability scores, and responses with more sentences were associated with higher scores, model differences persisted after controlling for these covariates. These data suggests that factors beyond length contribute to the observed differences in readability.

It is important to note, however, that Flesch–Kincaid scores—while useful for assessing surface-level readability—do not account for users’ actual comprehension of medical content. This metric evaluates only sentence length and syllable count, which may underestimate the difficulty of interpreting specialized clinical terms or nuanced health information. As a result, Flesch–Kincaid may fail to detect true barriers to understanding for individuals unfamiliar with medical vocabulary. This limitation underscores the need to pair quantitative readability assessments with qualitative evaluation methods, such as patient or clinician review, to better gauge true comprehensibility and usability of AI-generated content in healthcare settings. Despite these limitations, Flesch–Kincaid scores remain among the most widely used readability measures in both healthcare and NLP research, as noted above, making them appropriate for our study goal of comparing models on a standardized and reproducible basis.

### Similarity of NLP model responses to frequently asked CVD questions

Similarity is a measurement of the textual and contextual meaning of a set of written text. The similarity between response sets across models was insignificant with respect to education level. Accordingly, regardless of a user’s education, each NLP model provides comparable textual and contextual information in response to CVD queries. However, ChatGPT Free and ChatGPT Premium were found to be more similar to each other compared to MediSearch. In other words, ChatGPT Free and ChatGPT Premium exhibit greater similarity to each other than ChatGPT Free or ChatGPT Premium does to MediSearch. This is likely because both platforms within ChatGPT share a similar “knowledge cutoff” that will not allow either to generate responses beyond their respective training periods (33). While this may be intuitive, findings from the present investigation underscores the consistency of ChatGPT’s responses across education levels and model types, a key advantage for individuals who do not want or cannot afford ChatGPT Premium. In addition, response similarity differs significantly when comparing both ChatGPT models to MediSearch, likely due to its specialized focus on medical content and its unique response generation methodology. The absence of significant correlation between education level and similarity implies consistency in how each model relates to one another regardless of user educational background. This underscores inherent model-specific differences rather than differences driven by user educational factors. Although the similarity between ChatGPT Free and Premium appears intuitive, this analysis explicitly validates the assumption that different versions of a single NLP platform maintain consistent response patterns regardless of user education level. Demonstrating consistent similarity internally (between different versions of the same model) serves as a baseline for assessing divergence against other specialized medical NLP models like MediSearch. While the present study focused on readability and similarity, all responses were drawn directly from models without altering clinical content. Although formal accuracy scoring was not conducted, the responses were preserved exactly as they were generated to avoid bias. We recognize that accuracy influences readability and similarity, and propose future work integrating accuracy, readability, and similarity for more comprehensive evaluation. Such differences highlight the practical implication that selection of an NLP tool could significantly influence the nature of the healthcare information users receive. Nevertheless, future analyses could further explore whether higher or lower similarity correlates with readability outcomes, thereby strengthening the practical applicability of these analyses.

Data from the present investigation indicates that ChatGPT Premium can explain ~35% of the variance in the Flesch–Kincaid Ease Score of ChatGPT Premium responses; therefore, ChatGPT premium is the strongest predictor of readability when correlated with education level among all AI models. ChatGPT Free explains ~28% of the variance in readability across education levels, and MediSearch explains ~23%, making it the weakest predictor, although statistically significant (*p* < 0.001). Nonetheless, the findings from the present investigation indicate that individuals with higher education levels engage with the models in a more intricate way, leading to differences in how each model generates a response. An important implication of this finding is that NLP-model generated content could be considered more uniform and less tailored to nuanced inquiries from those with lower educational backgrounds, leading to concerns of oversimplification or misinterpretation. Conversely, the variability of responses at higher education levels may suggest that models struggle to provide consistent responses to more sophisticated questions, opening the door to inaccuracies and misinterpretation. It is important to emphasize that nuances in responses as a result of prompt input because this study used education level to assess readability, but education level may not necessarily correspond to medical literacy. Even though increased education generally corresponds to increased medical literacy (34), some people with advanced degrees may have low health literacy as input prompts at a lower level than their education would indicate.

The present findings are broadly consistent with prior investigations evaluating AI-generated cardiovascular and general medical information. Similar to the current study, Anaya et al. and Olszewski et al. found that ChatGPT responses about heart failure and cardiovascular topics exceeded recommended readability levels and often required college-level comprehension (7, 8). Dayı et al. also reported variability in chatbot accuracy and accessibility in coronary artery disease education (9), while Squizzato et al. observed inconsistent clarity of ChatGPT answers regarding cardiac arrest (10). Collectively, these studies—and the present analysis—demonstrate that large language models frequently produce linguistically complex content across specialties, underscoring the need for readability optimization and model fine-tuning. However, unlike these earlier works, our study uniquely stratifies results by user education level, offering a more nuanced understanding of how readability adapts (or fails to adapt) to user background.

### Future considerations

This study has several important limitations. First, although 35 cardiovascular FAQs were systematically reformulated into three education-level variants (Easy, Medium, and Hard), these grade-level classifications are only proxies. Real-world patient queries may not align with grade-level readability scores, and future work should incorporate authentic patient-generated questions. Second, a single-run design was employed for each prompt. While prior research suggests that responses from large language models are relatively stable across runs, variability is an inherent feature of these systems. Future studies should replicate prompts multiple times to evaluate output variance. Third, readability was assessed primarily using Flesch–Kincaid indices. These surface-level measures capture sentence length and word complexity but do not fully represent health literacy demands or comprehension of medical jargon. Although word and sentence count covariates were examined, lexical frequency analysis and clinician-based ratings would strengthen interpretability. Fourth, semantic similarity was measured with a transformer-based embedding model, which quantifies textual overlap but not clinical accuracy. Responses may be semantically similar while differing in correctness, underscoring the need for integrated evaluations of readability, similarity, and clinical appropriateness. Fifth, the study was limited by scope: only English-language queries were examined, focused on U.S.-based literacy standards, and based on a modest sample of 35 questions. Generalizability to other languages, healthcare contexts, and larger corpora remains uncertain. Finally, while emerging models such as DeepSeek and GPT-5 may represent future directions, this study did not evaluate their outputs directly. Ongoing work should assess both general-purpose and specialized medical models across multiple dimensions, including factual accuracy and patient comprehension.

### Future directions of AI in healthcare

There are many additional areas of exploration within the NLP space that can enhance researchers’ understanding of how these models interact with users. For example, future studies are needed to assess whether specialized medical AI models can be optimized to provide clearer, more accessible responses without compromising clinical accuracy. This could be investigated at the user level, understanding how more direct, yet simplified prompting can result in response changes. Additionally, future studies are needed to determine the specific effects that health literacy, rather than education level, has on NLP responses. Specifically, research should explore strategies to tailor prompts and responses based on user health literacy rather than solely on education level, ensuring that AI-generated medical content is both informative and comprehensible to a diverse population.

In assessing similarity, there also presents an opportunity to refine how we evaluate NLP model responses as semantic similarity does not equal clinical correctness. For example, two responses can be phrased similarly but have different clinical interpretations. While BERT-Cosine similarity is a valuable tool for estimating semantic overlap, it does not guarantee that two responses are equally accurate from a clinical perspective. This distinction is critical in medical applications, where subtle differences in language can significantly alter clinical meaning. Future studies should therefore consider incorporating subjective evaluations by clinicians or laypersons alongside objective metrics to ensure that AI-generated responses are not only similar in phrasing but also consistent with best clinical practices. Such triangulation could enhance confidence in the clinical appropriateness and safety of these tools.

The data collection for this study was conducted between August and December 2024. During this period, ChatGPT models—including GPT-4o—were subject to ongoing background updates, which may influence the consistency of responses over time. These updates, while often undocumented in granular detail, can lead to changes in model behavior, tone, and accuracy. As such, findings reported here represent a snapshot in time and may not fully reflect current or future model performance. This presents an inherent limitation in evaluating commercial NLP tools whose parameters may evolve without transparent version control. Future investigations should consider periodic re-evaluation of these models to monitor changes in response quality and clinical reliability. Additionally, while other widely used AI tools, such as Gemini, Claude, or Bing Health, could provide additional insights, the present study focused specifically on ChatGPT and MediSearch because these models were most prominently used in healthcare communication at the time of study initiation. Logistical considerations, such as availability, widespread adoption, and model maturity at the study’s commencement, guided our selection. Future research should incorporate additional AI tools to enhance external validity and provide broader generalizability. Importantly, Chat GPT-5 was not used for comparison because it was released after the data was collected for the present investigation (August–December 2024). To preserve methodological consistency, comparisons were limited to the three models that were widely available and stable at the time of study initiation.

Lastly, it is important to note that AI-driven tools are rapidly evolving, and new models continue to emerge. While this study specifically examined ChatGPT and MediSearch, future work should periodically re-evaluate these and other models to ensure findings remain relevant as the technology advances.

## Conclusion

In conclusion, findings from the present investigation highlight the potential of AI-driven NLP models, such as ChatGPT and MediSearch, to adapt responses based on user education level. However, data also supports that challenges in balancing readability and complexity to AI-generated responses to cardiovascular disease queries exist. While all models examined (ChatGPT Free, ChatGPT Premium, and MediSearch) adapt to user education levels, the overall complexity of response remains high, often exceeding the comprehension level of individuals without a college education. ChatGPT Free demonstrated the strongest adaptability to education level, whereas the least variation was observed using MediSearch, suggesting that general-purpose models may offer more accessible health information than specialized medical AI tools. Moving forward, ensuring that AI-driven healthcare information is both accurate and comprehensible will be essential in making these tools truly beneficial for the public.

## Data Availability

The raw data supporting the conclusions of this article will be made available by the authors, without undue reservation.
